# Evaluation of enhanced external counterpulsation with different modes on acute hemodynamic effects

**DOI:** 10.3389/fphys.2025.1555358

**Published:** 2025-04-30

**Authors:** Yujia Zhong, Liling Hao, Xue Jia, Songrim Paek, Shuai Tian, Guifu Wu

**Affiliations:** ^1^ College of Medicine and Biological Information Engineering, Northeastern University, Shenyang, Liaoning, China; ^2^ Department of Cardiology, The Eighth Affiliated Hospital of Sun Yat-sen University, Shenzhen, Guangdong, China

**Keywords:** enhanced external counterpulsation, cardiovascular disease, hemodynamic effects, blood flow, 0D–1D cardiovascular system model

## Abstract

**Objective:**

Enhanced external counterpulsation (EECP) is a noninvasive device for the treatment of cardiovascular diseases. However, there are minimal data regarding the effects of different EECP modes on acute hemodynamic changes, particularly blood flow redistribution. This study aimed to investigate the systemic hemodynamic effects during different EECP modes based on clinical trials and numerical analysis.

**Methods:**

Fifteen patients with cardiovascular disease and 15 healthy subjects completed four and six EECP modes, respectively. These EECP modes changed the parameters, including counterpulsation pressure, start time, and counterpulsation frequency. Hemodynamic parameters in the aorta (AO), right femoral artery (RF), and right brachial artery (RB), including mean flow rate (FR), mean blood velocity (MV), peak systolic velocity (PSV), minimum diastolic velocity (MDV), and diastolic/systolic blood pressure ratio (D/S), were measured during EECP treatments. Meanwhile, the simulation of hemodynamic responses to different EECP modes based on a 0D-1D cardiovascular system model were conducted and compared with the clinical results.

**Results:**

As counterpulsation pressure increased, the FR and PSV of AO, the FR, MV, PSV, and MDV of RF, the FR, MV, and MDV of RB, and D/S increased in patients (*all P < 0.05*). The MV of RF, the FR, MV, PSV, and MDV of RB, and D/S of patients decreased significantly with increasing start time (*all P < 0.05*). For the increase of counterpulsation frequency, the FR, MV, and PSV of AO, the MV, PSV, and MDV of RF, and the FR and MV of RB significantly decreased in patients (*all P < 0.05*). For the health group, most patients’ results were similar. Multiple groups of pressure experiments indicated that 25–30 kPa significantly improved blood flow. The numerical results under different EECP modes were generally closely aligned with clinical measurements.

**Conclusion:**

Different EECP modes induced different hemodynamic responses. Higher counterpulsation pressure, T wave start time, and 1:1 counterpulsation frequency are recommended to improve blood flow. Hemodynamic simulations prepare the way for the creation of virtual databases to obtain population-based strategies and then allow for precision-based strategies through individual modeling. The different hemodynamic responses to EECP modes provide theoretical guidance for the development of a patient-specific treatment strategy.

## 1 Introduction

Enhanced external counterpulsation (EECP) is a kind of auxiliary circulation method which employs electrocardiogram-gated sequential inflation and deflation of cuffs wrapped around the calves, thighs, and buttocks (Soubh et al., 2023). At the beginning of the diastole, the cuffs inflate and apply pressure to blood vessels in the lower limbs, increasing diastolic blood pressure and blood flow back to the heart. Then, the cuffs deflate quickly, reducing the systolic blood pressure to make it easier for the heart to pump again (Qin et al., 2016). The technique promotes blood flow redistribution, improving organ ischemia by increasing blood flow in the aorta, cerebral artery, carotid artery, hepatic artery, femoral artery, and other regions (Zeng et al., 2024; Li et al., 2019). EECP was first introduced in the chronic stable angina therapy approved by the Food and Drug Administration (FDA), which offers a safe, noninvasive and effective therapy for patients who are unresponsive to other treatments or not eligible for surgery (Ashokprabhu et al., 2024; Gurovich and Braith, 2013). Clinical findings have demonstrated that EECP also plays beneficial roles, including reducing the Canadian Cardiovascular Society (CCS) angina classification, lowering advanced glycation end-product (AGE) concentrations in patients with type 2 diabetes mellitus, and improving ocular blood flow and visual function in patients with non-arteritic anterior ischemic optic neuropathy (NAION) (Ashokprabhu et al., 2024; Sardina et al., 2016; Zhu et al., 2015; Xu et al., 2024).

EECP has been shown to increase blood flow in the aortic root, elevate diastolic pressure, lower systolic pressure, and enhance shear stress (Xu et al., 2023). Increase in shear stress promotes endothelial cell differentiation while inhibiting excessive proliferation and apoptosis (Gijsen et al., 2019). Additionally, endothelial cell growth modulates the secretion of vasoactive substances, thereby promoting coronary artery dilation and improving endothelial function (Braith et al., 2012; Braith et al., 2010). Some studies also suggest that shear stress is associated with the regulation of inflammatory factors, adhesion molecules, and oxidative stress (Braith et al., 2012), which slows the progression of atherosclerosis, stabilizes plaques, and reduces the occurrence of acute coronary events (Xu et al., 2023). Moreover, EECP has been shown to improve the collateral flow index (CFI) and fractional flow reserve (FFR), enhancing collateral circulation and myocardial perfusion (Buschmann et al., 2009). Following EECP treatment, improvements have been observed in heart failure patients, including increased left ventricular ejection fraction, decreased left ventricular end-systolic volume, improved aortic wave reflection time and amplitude, reduced myocardial oxygen demand index (Casey et al., 2011), and enhanced exercise capacity. These effects contribute to better myocardial oxygen supply and reduced myocardial workload. Therefore, the beneficial effects of EECP are associated with improvements in both flow and velocity.

At present, most research works on EECP focus on evaluating the therapeutic improvement of diseases such as coronary artery disease and other ischemic conditions. However, EECP is operated with manual adjustment by clinical experience, which cannot guarantee treatment efficacy and is known to cause discomfort in patients. The hemodynamic effects of different EECP modes remain uncertain. This may hinder the improvement and development of EECP, reduce the accuracy and efficiency of clinical treatment, and potentially lead to misinterpretations of therapeutic effects due to improper EECP modes. Abdelwahab and Elsaied (2018) and Yavari and Montazeri (2007) set counterpulsation pressure at 220–250 mmHg. Although significant improvements in angina frequency were observed after 7 weeks, there was no notable improvement in left ventricular ejection fraction (LVEF). Conversely, in Kozdağ et al. (2012), where the counterpulsation pressure was increased to 280–300 mmHg, significant improvements in LVEF were observed after 7 weeks. Although differences in patient populations may affect these outcomes, these findings still highlight the potential impact of adjusting the EECP modes. Therefore, the hemodynamic effects of EECP modes should be explored more comprehensively.

Nowadays, there are two approaches in the study of the hemodynamic effects of different EECP modes, clinical methods, and model-based methods. In clinical studies, researchers have explored the influence of varying EECP modes on local hemodynamic or biochemical parameters (Xiong et al., 2015; Lin et al., 2013; Coombes et al., 2023; Zhang et al., 2021). For example, Jeff S. measured HbA1c levels in subjects with type 2 diabetes by varying the duration of each EECP treatment (Coombes et al., 2023). The results indicated that after 7 weeks of 20 sessions of 45-min EECP, blood glucose control improved significantly more than in the 20 sessions of 30-min treatments or the control group that did not receive EECP therapy. Zhang et al. explored the impact of different compression site combinations on lower limb circulation. Their findings revealed that EECP-3 (involving the calves, thighs and buttock) significantly enhanced blood flow in the anterior tibial artery, and the other two modes resulted in significant increases in blood flow velocity of the inferior knee artery (Zhang et al., 2021). However, these clinical trials were limited by small sample size studies or few vascular sites, resulting in clinical conclusions that cannot be generalized to larger population. Thus, larger and more multifaceted studies should be conducted to strengthen these results.

Therefore, model-based research for the hemodynamic effects of different EECP modes emerged to address the problems of clinical trials, such as complex-to-measure hemodynamic parameters at all the sites of interest, *in vivo* measurements subject to experimental error, and being expensive and time-consuming. Model-based methods can not only investigate the population-based strategy based on *in silico* comprehensive datasets but also can conduct patient-specific treatment based on the personalized hemodynamic model. These model-based methods save resources, reduce the risk of clinical trials, and help improve clinical benefits. At present, model-based research of different EECP modes is carried out through the personalized hemodynamic models. Some researchers designed hemodynamic models to simulate various EECP modes and analyze blood flow parameters to evaluate the impact of these modes (Zhang X. C. et al., 2024; Li et al., 2021; Ye et al., 2022; Du et al., 2023; Chen et al., 2023; Li et al., 2023). Ye et al. combined EECP with a lumped parameter model, verifying that increasing the frequency of EECP can improve blood perfusion (Ye et al., 2022). Du et al. developed a 3D numerical model, demonstrating the positive effect of increased counterpulsation pressure on improving lower extremity circulation (Du et al., 2023). Chen et al. established 0D/3D geometric multi-scale hemodynamic models and found that coronary artery flow, cerebral blood flow and area-time-averaged wall shear stress in the coronary and cerebral arteries were higher when one counterpulsation was applied per cardiac cycle compared to two or three cardiac cycles (Chen et al., 2023). Li et al. coupled a 0D-3D geometric multi-scale hemodynamic cerebral artery model with automatic regulation and found that longer pressurization times should be chosen for EECP treatment in patients with more severe stenosis caused by cerebral ischemic stroke to achieve better therapeutic effects (Li et al., 2023). These simulation studies focused on the construction of hemodynamic simulation models and conduct simulation by changing one EECP parameter. In addition, compared to the local hemodynamic effects, the global hemodynamic effects of different EECP modes, particularly blood flow redistribution, have yet to be studied.

Overall, there are minimal data regarding the effects of different EECP modes on acute hemodynamic changes, particularly blood flow redistribution. In the present study, we investigated systemic hemodynamic effects during different EECP modes, including different counterpulsation pressures, start times, and counterpulsation frequencies, based on clinical trials and numerical analysis. For clinical trials, the acute effects of different EECP modes on the hemodynamics of the aorta, right brachial artery, and right femoral artery were investigated in both patients with cardiovascular disease and in healthy subjects. However, clinical trials cannot measure hemodynamic information at all locations, and more detailed studies of EECP modes are often limited by clinical costs and time constraints. In contrast, simulation studies can effectively overcome these limitations. Therefore, this study conducted a simulation analysis based on clinical experiments to validate the effectiveness of the simulation model. The hemodynamic responses to the different EECP modes were simulated using our previously developed 0D-1D cardiovascular system model which can provide accurate hemodynamic quantities in any blood vessel (Zhang et al., 2022). The clinical and simulation results can confirm each other. This study can clarify the acute responses of these EECP modes on systemic hemodynamics, and it may provide theoretical guidance for developing a patient-specific treatment strategy.

## 2 Materials and methods

### 2.1 Subjects

We conducted recruitment at the Cardiovascular Medicine Department of the Eighth Affiliated Hospital of Sun Yat-sen University, enrolling a group of patients diagnosed with coronary artery disease (CAD) for participation (n = 15) in EECP therapy. Meanwhile, we assembled a group of healthy subjects (n = 15) recruited from the Health Examination Center of the Eighth Affiliated Hospital of Sun Yat-sen University. Table 1 shows the detailed information of the subjects. All the participants signed written informed consent forms. The experiments were approved by the Institutional Review Board of the Eighth Affiliated Hospital of Sun Yat-sen University (registration number 2021-020-02).

**TABLE 1:** Clinical characteristics of the subjects and the treatment plan (n = 30).

CAD patients (n = 15)			Healthy subjects (n = 15)		
Index	Range	Mean ± SD	Index	Range	Mean ± SD
Age (years old)	[50, 81]	59.4 ± 10.6	Age (years old)	[19, 33]	24.0 ± 4.4
Height (cm)	[155, 180]	167.1 ± 8.2	Height (cm)	[157, 185]	172.8 ± 7.8
Weight (kg)	[51, 82]	69.3 ± 8.6	Weight (kg)	[43, 90]	64.1 ± 11.7
Number of men	11	—	Number of men	13	—
Number of women	4	—	Number of women	2	—
SBP (mmHg)	[113, 160]	130 ± 15.5	SBP (mmHg)	[102, 125]	112.5 ± 8.3
DBP (mmHg)	[66, 95]	70 ± 9.0	DBP (mmHg)	[53, 83]	70.0 ± 6.8
HR (bpm)	[50, 83]	64.2 ± 10.0	HR (bpm)	[57, 99]	71.1 ± 10.5
Rest	0 kPa		Rest	0 kPa	
EECP-P30	30 kPa, T wave, 300 ms, 1:1		EECP-P30	30 kPa, T wave, 300 ms, 1:1	
EECP-P20	20 kPa, T wave, 300 ms, 1:1		EECP-P20	20 kPa, T wave, 300 ms, 1:1	
EECP-Time	30 kPa, T wave +0.2T, 300 ms, 1:1		EECP-Time	30 kPa, T wave +0.2T, 300 ms, 1:1	
EECP-Freq	30 kPa, T wave, 300 ms, 1:2		EECP-Freq	30 kPa, T wave, 300 ms, 1:2	
EECP-P25	—		EECP-P25	25 kPa, T wave, 300 ms, 1:1	
EECP-P35	—		EECP-P35	35 kPa, T wave, 300 ms, 1:1	

SBP, systolic blood pressure; DBP, diastolic blood pressure.

### 2.2 Experiment scheme

**FIGURE 1:**
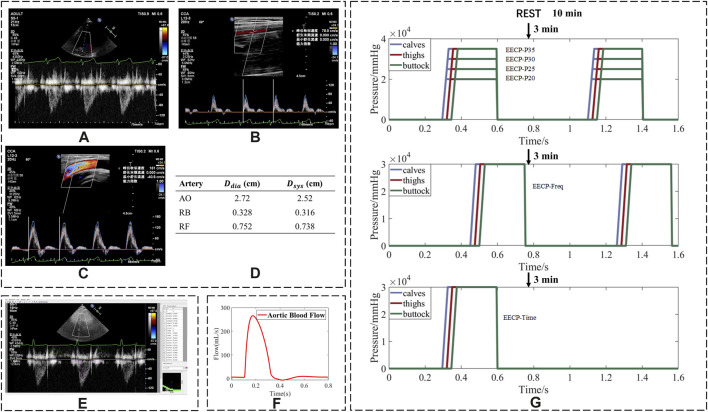
Process of converting ultrasonic images into flow rates and experiment sequence of EECP. Ultrasound images of **(A)** aorta; **(B)** right brachial artery; **(C)** right femoral artery; **(D)** diameter of arteries; **(E)** using Getdata software to trace the spectrum of blood flow velocity; **(F)** blood flow waveform after smoothing; **(G)** flowchart of the whole experimental scheme (assuming a cardiac cycle duration of 0.8 s).

Before the experiment, participants were required to maintain a normal physiological state for at least 4 h after eating. The experiments were performed at the same time of each day (5:00 p.m.–7:00 p.m.). All subjects were first asked to undergo a review to confirm that there were no contraindications for EECP, and basic information about the subjects was recorded (Table 1). Electrodes were then attached to the subject’s body surface, as the range of EECP is determined by electrocardiogram (ECG), and cuffs were wrapped around the lower extremities to squeeze the vessels of the lower limbs. The subjects’ blood flow rates were measured both at rest and during EECP states with varying modes, while blood pressure was measured only at rest. Blood pressure was measured using an electronic sphygmomanometer (Omron U724). The measurements were performed for each group in the supine position after 10 min of relaxation. All subjects received EECP intervention with the PSK P-ECP/TM Oxygen Saturation Monitoring EECP Instrument (made in Chongqing, China). The detailed measurement process is shown in Figure 1G and Table 1. We collected ultrasound data at the aorta (AO), the right brachial artery (RB), and the right femoral artery (RF) using an ultrasound device (Philips EPIQ 7) with cardiac synchronization. Measurements were taken by a sonographer, who adjusted the probe angle to achieve optimal ultrasound signal quality and strength. Every effort was made to ensure consistency across all measurements, with particular attention paid to the precise positioning of blood vessels. To ensure accuracy, multiple cardiac cycles were measured.

In a few cases, the lengthy duration of the ultrasound measurement could lead to mild discomfort for the patients. Therefore, the data were collected in the CAD group for only five states (Table 1). For the healthy group, the data were measured in sequence for seven states (Table 1).

### 2.3 Parameter calculation

The collected ultrasound images are shown in Figures 1A–C. Peak systolic velocity (PSV) and minimum diastolic velocity (MDV) parameters were recorded. GetData Graph Digitizer® software was used to derive the blood flow velocity curve from the ultrasonic blood flow velocity spectrum image (Figure 1E) and obtain the mean blood velocity (MV). The velocity parameters are described in absolute terms.

The blood flow curve in Figure 1F was calculated from the blood velocity curve, and the internal diameter was measured by ultrasound (Figure 1D). The mean flow rate (FR) was calculated from the blood flow curve. In addition, the values of diastolic/systolic blood pressure ratio (D/S) displayed on the screen of the EECP instrument were also recorded. The ratios of FR, MV, PSV, and MDV were calculated by dividing their values in the EECP state by those in the rest state.

### 2.4 Statistical analysis

The results were represented as mean ± SD to show the central tendency of the data and its degree of dispersion. All hemodynamic parameters except D/S were expressed as the ratio of EECP states to rest state. A Kolmogorov–Smirnov test was used to evaluate the normal distribution of hemodynamic variables (at least one test p > 0.05). The basic characteristics of hemodynamic variables were then determined by descriptive statistical analysis. For comparisons of different modes in the same group, a paired t-test (between two modes) or repeated-measures ANOVA (among four modes) was utilized. Independent samples t-tests comparing hemodynamic parameters between CAD patients and healthy subjects in the same EECP mode were performed. The *post hoc analysis* used least-significant difference. In this study, the effect size was calculated using eta squared (η^2^), which represents the proportion of variance in the dependent variable explained by the independent variable. The magnitude of the effect size was interpreted as small (η^2^ ≥ 0.01), medium (η^2^ ≥ 0.06), or large (η^2^ ≥ 0.14) based on Cohen’s guidelines. All statistical tests were performed using SPSS version 27.0.1 (IBM SPSS Statistics, Chicago, IL, United States); p < 0.05 was considered statistically significant.

### 2.5 0D–1D cardiovascular system model

**FIGURE 2:**
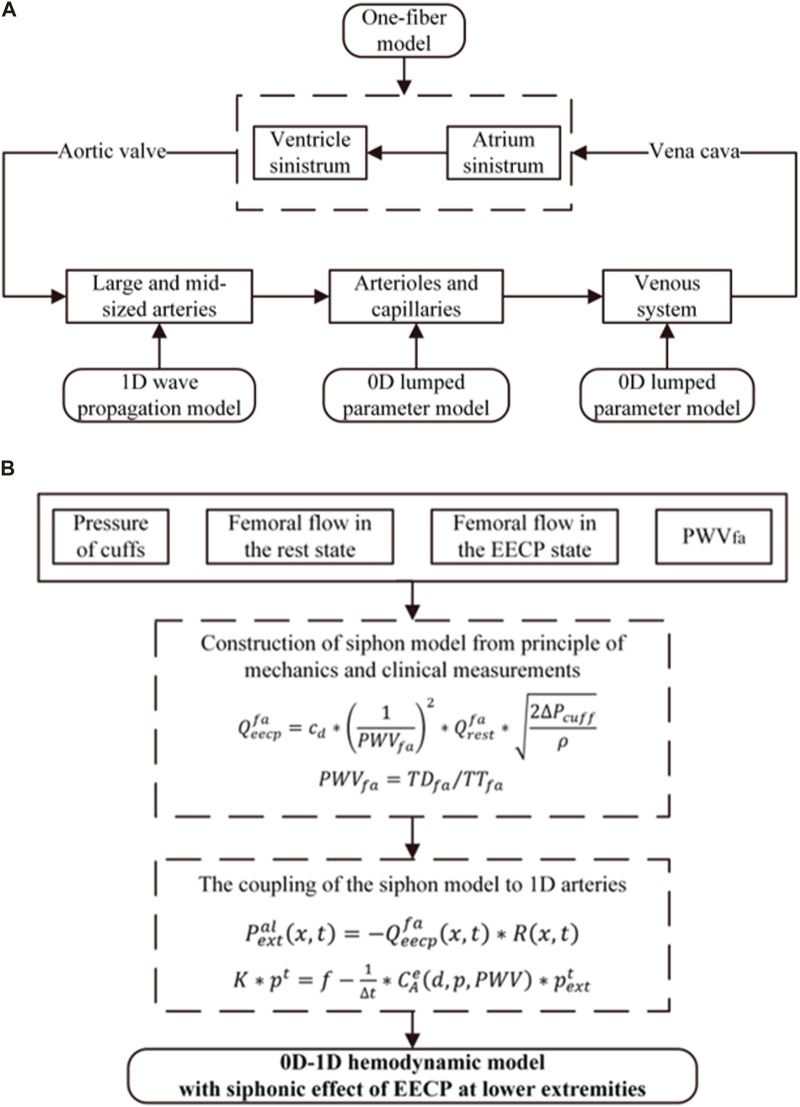
**(A)** Structure of the closed-loop 0D-1D model of the cardiovascular system. **(B)** EECP model coupled with 0D-1D hemodynamic model.

A closed-loop 0D–1D model comprising a series of components to accurately represent the hemodynamic characteristics of the circulation system was constructed based on our previous research (Zhang et al., 2022). As shown in Figure 2A, the 0D-1D model of the cardiovascular system encompassed the heart (0D one-fiber model), 55 arteries (1D wave propagation model), arterioles and capillaries (0D model), and a venous vascular tree (0D model). After being ejected from the left ventricle, blood enters the aorta, flows through the arterial system, reaches the arterioles and capillaries, enters the venous system, returns to the right atrium via the superior and inferior vena cava, and finally flows to the left ventricle, completing a continuous closed-loop cycle.

The numerical calculation of the siphon model refers to our previous research, where $Q_{eecp}^{fa}$ is the femoral arterial blood flow under EECP state, $Q_{rest}^{fa}$ is the femoral arterial blood flow under rest state, ${∆P}_{cuff}$ is the difference between the central arterial pressure and the counterpulsation pressure, ${TD}_{fa}$ is 0.375 times the height, and ${TT}_{fa}$ denotes the aorta–femoral transit time.

### 2.6 EECP model

The EECP effects coupled to the 1D arterial tree are shown in Figure 2A. For the 1D model, the extra-membrane pressure induced by EECP acts through the local area compliance of the artery, which is the product of the pressure-dependent function and a location-dependent function. Therefore, the pulse wave velocity, which is directly associated with the vascular stiffness, is identified as the critical parameter for coupling the EECP model to 1D model. The EECP model combined with the 1D model has higher accuracy and wider applicability. Additionally, it can explain the different EECP effects on subjects with different levels of vascular stiffness. The effect of EECP on the 0D vein model was considered; when simulating the vascular collapse caused by EECP, the compliance was ignored in the vein model. Detailed methods can be found in our previous research (Zhang et al., 2022).

EECP improves blood circulation through sequential mechanical compression on lower limbs, so the accuracy of hemodynamics in the lower extremity arteries plays a crucial role in determining the effectiveness of EECP treatment. In our previous research, a siphon model was developed to investigate the interdependence of several hemodynamic parameters, including pulse wave velocity, femoral flow rate, the pressure of cuffs, and the mean blood flow changes in the femoral artery throughout EECP therapy (Zhang Q. et al., 2024). The siphon model integrated with the 0D-1D model can estimate blood flow profiles under varying EECP pressures. In this study, the improved siphon model was developed to determine the relationship between the varying EECP start times and the hemodynamic changes in the mean femoral blood flow. This model was parameterized using four variables: the pressure of cuffs, mean femoral flow during both rest and EECP, and pulse wave velocity. Finally, the improved siphon model was coupled with the 0D-1D model to simulate hemodynamic responses to the EECP treatment at different EECP start times. The flowchart of this study is shown in Figure 2B.

## 3 Results

Table 2 summarizes the hemodynamic parameters of CAD patients and healthy subjects under both rest state and EECP state, with values reported as mean ± SD.

**TABLE 2:** The hemodynamic parameters (mean ± SD) in the rest and EECP states.

CAD patients		Rest	EECP-P30	EECP-P20	EECP-time	EECP-freq
Aortic	FR (mL/min)	11,439 ± 5,526	12,623 ± 5,374	11,433 ± 4,071	11,554 ± 4,081	10,292 ± 3,820
	MV (cm/s)	28.0 ± 6.4	31.3 ± 5.4	29.7 ± 4.9	29.2 ± 6.6	28.3 ± 5.5
	PSV (cm/s)	113.2 ± 18.0	121.9 ± 21.4	112.8 ± 17.0	115.4 ± 16.9	114.3 ± 18.0
	MDV (cm/s)	−16.6 ± 3.3	−16.6 ± 2.9	−16.9 ± 2.6	−16.7 ± 2.4	−16.4 ± 2.9
Femoral	FR (mL/min)	254.1 ± 106.1	464.6 ± 316.7	325.5 ± 210.9	499.1 ± 243.4	423.9 ± 355.1
	MV (cm/s)	8.4 ± 3.5	13.1 ± 6.4	9.8 ± 5.4	13.1 ± 5.2	11.4 ± 7.4
	PSV (cm/s)	118.1 ± 26.5	248.5 ± 56.7	156.5 ± 38.9	267.5 ± 63.7	202.4 ± 45.1
	MDV (cm/s)	−40.9 ± 9.2	−199.8 ± 50.5	−102.7 ± 35.6	−217.9 ± 57.0	−147.0 ± 70.5
Brachial	FR (mL/min)	66.6 ± 35.4	85.7 ± 35.9	73.5 ± 32.0	68.2 ± 32.0	68.2 ± 30.5
	MV (cm/s)	14.9 ± 4.2	56.4 ± 20.8	31.2 ± 10.9	46.5 ± 16.3	28.9 ± 10.2
	PSV (cm/s)	76.7 ± 17.9	85.2 ± 28.4	83.9 ± 29.7	76.2 ± 27.4	83.3 ± 26.2
	MDV (cm/s)	−16.3 ± 7.6	−18.6 ± 6.6	−16.6 ± 5.2	−22.7 ± 6.3	−22.1 ± 8.3

Healthy subjects		Rest	EECP-P30	EECP-P20	EECP-Time	EECP-Freq	EECP-P25	EECP-P35
Aortic	FR (mL/min)	7,280 ± 3,195	7,681 ± 2,603	6,822 ± 2048	7,068 ± 2,600	5,968 ± 1,490	7,428 ± 2,529	7,825 ± 2,777
	MV (cm/s)	23.8 ± 6.3	24.0 ± 5.0	22.4 ± 4.9	22.9 ± 4.2	21.2 ± 3.3	22.7 ± 4.4	24.0 ± 5.1
	PSV (cm/s)	102.1 ± 14.9	107.9 ± 18.5	103.4 ± 16.7	106.7 ± 15.1	101.8 ± 14.1	97.8 ± 11.2	103.3 ± 15.7
	MDV (cm/s)	−17.6 ± 3.2	−20.2 ± 3.8	−21.1 ± 4.6	−18.8 ± 4.0	−18.7 ± 1.9	−19.1 ± 3.1	−17.7 ± 3.7
Femoral	FR (mL/min)	417.4 ± 221.1	922.5 ± 573.4	573.1 ± 378.5	702.2 ± 408.5	512.9 ± 361.6	575.0 ± 529.8	938.2 ± 523.1
	MV (cm/s)	16.2 ± 5.8	73.4 ± 27.5	50.8 ± 17.3	65.6 ± 20.3	36.5 ± 12.6	56.2 ± 19.9	72.7 ± 22.5
	PSV (cm/s)	109.9 ± 31.5	312.3 ± 48.9	220.7 ± 47.0	312.7 ± 78.1	264.4 ± 65.8	262.9 ± 62.0	335.1 ± 64.8
	MDV (cm/s)	−38.2 ± 8.5	−250.2 ± 58.5	−172.1 ± 48.6	−231.0 ± 66.2	−259.2 ± 56.0	−218.8 ± 41.7	−286.4 ± 76.5
Brachial	FR (mL/min)	114.7 ± 66.3	108.8 ± 59.3	77.5 ± 41.2	110.6 ± 74.0	86.2 ± 38.1	106.2 ± 76.7	93.7 ± 63.5
	MV (cm/s)	13.8 ± 5.4	16.7 ± 3.5	11.9 ± 3.2	15.3 ± 5.8	12.3 ± 3.1	14.7 ± 5.29	15.9 ± 4.5
	PSV (cm/s)	99.6 ± 20.6	100.2 ± 30.2	91.3 ± 21.5	86.8 ± 21.8	99.9 ± 24.9	93.2 ± 20.2	101.4 ± 31.9
	MDV (cm/s)	−21.2 ± 8.6	−26.6 ± 18.0	−22.2 ± 8.4	−24.1 ± 11.0	−20.6 ± 10.8	−23.3 ± 11.3	−27.8 ± 9.6

### 3.1 Different EECP modes for CAD patients

#### 3.1.1 Counterpulsation pressure

**FIGURE 3:**
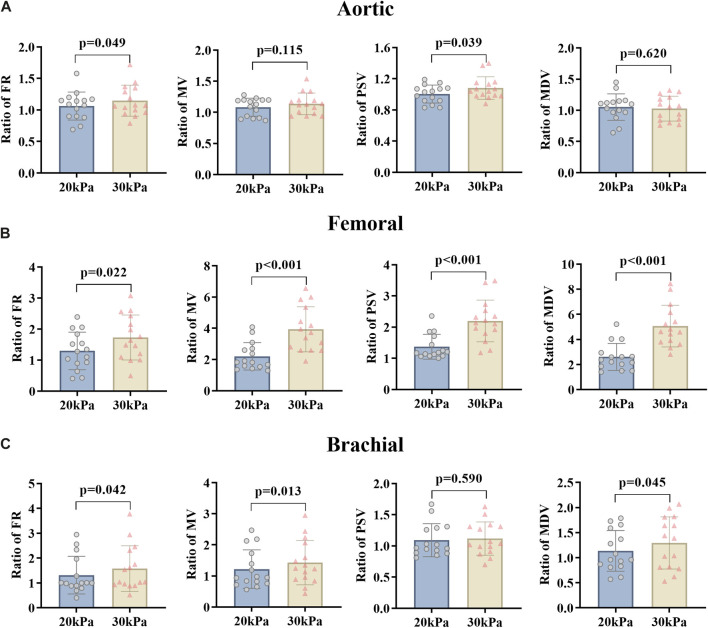
FR, MV, PSV, and MDV ratio of EECP-P20 (20 kPa, T wave, 1:1) and EECP-P30 (30 kPa, T wave, 1:1) of AO **(A)**, RF **(B)**, and RB **(C)** in the patient group. Results were evaluated by the paired-sample t-test.

The effects of the counterpulsation pressure in two states (EECP-P30 and EECP-P20) on the hemodynamic parameters of AO, RF, and RB in CAD patients are shown in Figure 3. The results show that the EECP-P30 and EECP-P20 are significantly different in the FR (p = 0.049) and PSV (p = 0.039) of AO. Meanwhile, FR, MV, PSV, and MDV were more significantly proportionally elevated with increasing counterpulsation pressure in the RF (*all P < 0.05*). FR (p = 0.042), MV (p = 0.013), and MDV (p = 0.045) were more significantly elevated proportionally with increasing counterpulsation pressure in the RB.

#### 3.1.2 Start time of counterpulsation

**FIGURE 4:**
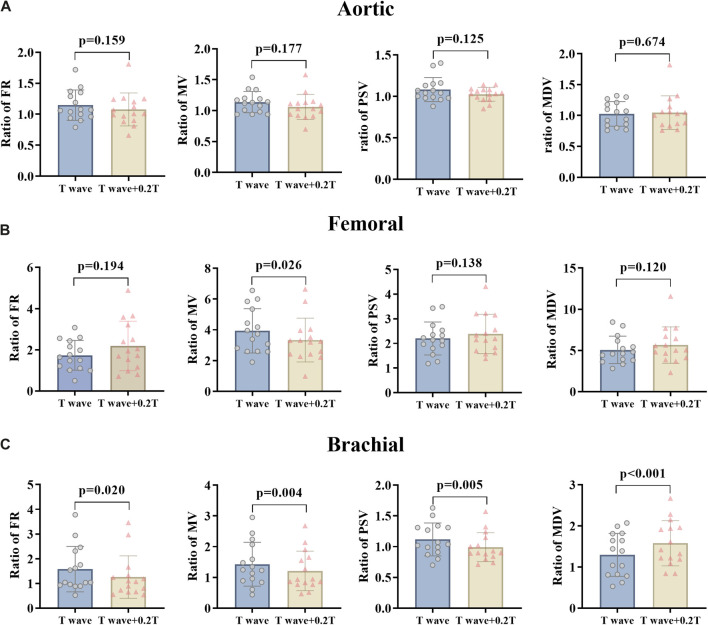
FR, MV, PSV, and MDV ratio of EECP-P30 (30 kPa, T wave, 1:1) and EECP-Time (30 kPa, T wave + 0.2 T, 1:1) of AO **(A)**, RF **(B)**, and RB **(C)** in the patient group. Results were evaluated by paired-sample t-test.

For changing the start time of counterpulsation, the hemodynamic parameters of AO, RF, and RB in the patient group were severally compared between the EECP-P30 with pressurization at the T wave and the EECP-Time with pressurization after the T wave with 20% of the cardiac cycle (Figure 4). The results showed no significant differences in the AO. At the same time, in the RB, FR (p = 0.020), MV (p = 0.004), and PSV (p = 0.005) were reduced significantly, while MDV increased significantly (p < 0.001). In the RF, MV was significantly different (p = 0.026).

#### 3.1.3 Counterpulsation frequency

**FIGURE 5:**
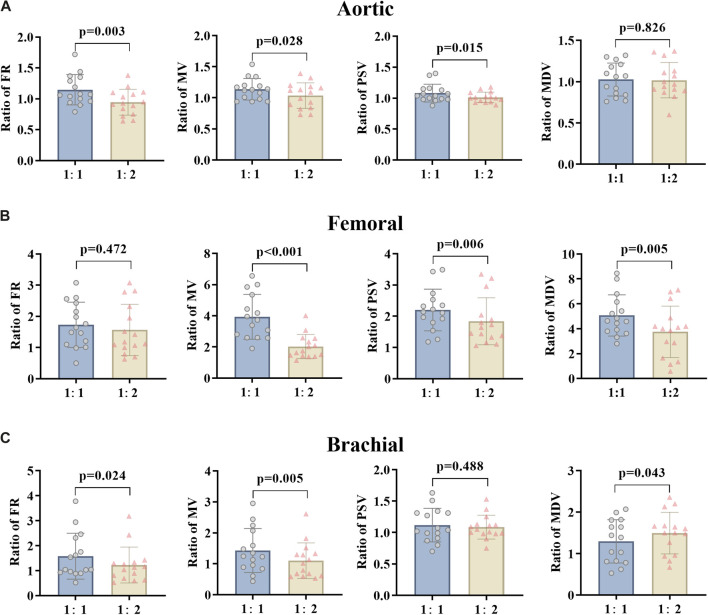
FR, MV, PSV, and MDV ratio of EECP-P30 (30 kPa, T wave, 1:1) and EECP-Freq (30 kPa, T wave, 1:2) of AO **(A)**, RF **(B)**, and RB **(C)** in the patient group. Results were evaluated by paired-sample t-test.

By changing the counterpulsation frequency in CAD patients, the hemodynamic parameters of the AO, RF and RB were counted, comparing EECP-P30 (counterpulsation frequency 1:1) with EECP-Freq (counterpulsation frequency 1:2) (Figure 5). This showed that the FR, MV, and PSV of AO and the FR and MV of RB were significantly higher at the counterpulsation frequency of 1:1 than at 1:2 (*all P < 0.05*). At the same time, the MV (p < 0.001), PSV (p = 0.006), and MDV (p = 0.005) of the RF were reduced significantly. However, particularly for the MDV of the RB, the counterpulsation frequency of 1:2 showed a greater improvement than that of 1:1 (p = 0.043).

**FIGURE 6:**
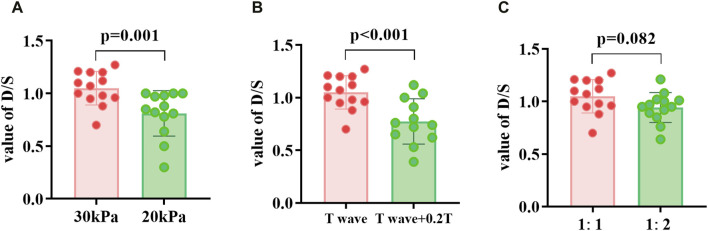
D/S value of EECP-P30 (30 kPa, T wave, 1:1) vs. EECP-P20 (20 kPa, T wave, 1:1) **(A)**, vs. EECP-Time (30 kPa, T wave +0.2T, 1:1) **(B)**, and vs. EECP-Freq (30 kPa, T wave, 1:2) **(C)** in patient group. Results were evaluated by paired-sample t-test.

The D/S values in the three groups of EECP states with different modes were compared (Figure 6). When the counterpulsation pressure increased or the start time decreased, the D/S value significantly increased (*P < 0.05*). However, no significant differences were observed when comparing different counterpulsation frequencies. Additionally, there were no significant differences in the internal diameters of vessels or heart rates across different states (EECP-P20 vs EECP-P30; EECP-P30 vs EECP-Time; EECP-P30 vs EECP-Freq).

### 3.2 Comparison of patient and healthy subjects

**FIGURE 7:**
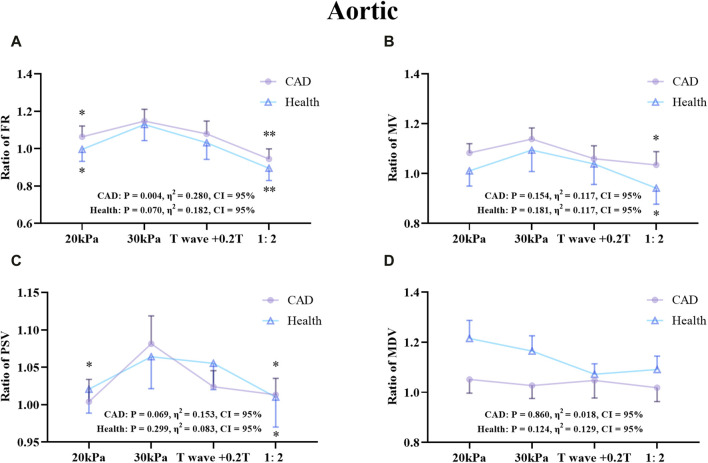
Comparison of ratio of FR **(A)**, MV **(B)**, PSV **(C)**, MDV **(D)** in the AO between the patient and healthy groups, *P < 0.05 EECP-P30 vs another EECP state, **P < 0.01 EECP-P30 vs another EECP state, ***P < 0.001 EECP-P30 vs. another EECP state. Results were evaluated by repeated-measures ANOVA (among 4 modes) by independent samples t-test (among two groups).

**FIGURE 8:**
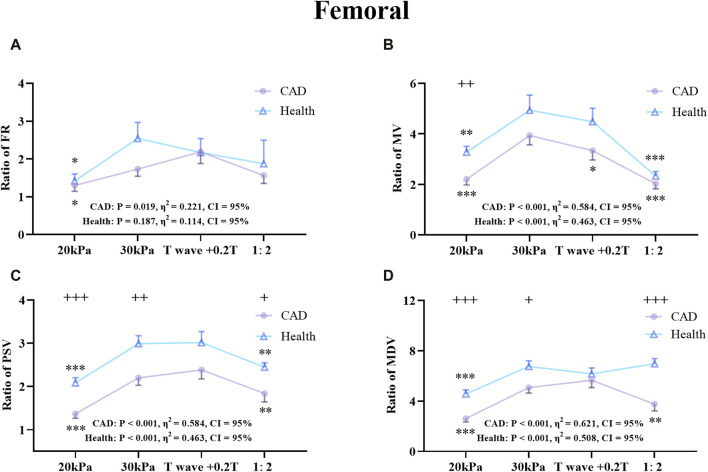
Comparison of the ratio of FR **(A)**, MV **(B)**, PSV **(C)**, MDV **(D)** in the RF between the patient and healthy group, *P < 0.05 EECP-P30 vs another EECP state, **P < 0.01 EECP-P30 vs another EECP state, ***P < 0.001 EECP-P30 vs. another EECP state, ^+^ *P* < 0.05 CAD vs Healthy, ^++^ *P* < 0.01 CAD vs. Healthy, ^+++^ *P* < 0.001 CAD vs Healthy. Results were evaluated by repeated-measures ANOVA (among 4 modes) by independent samples t-test (among 2 groups).

**FIGURE 9:**
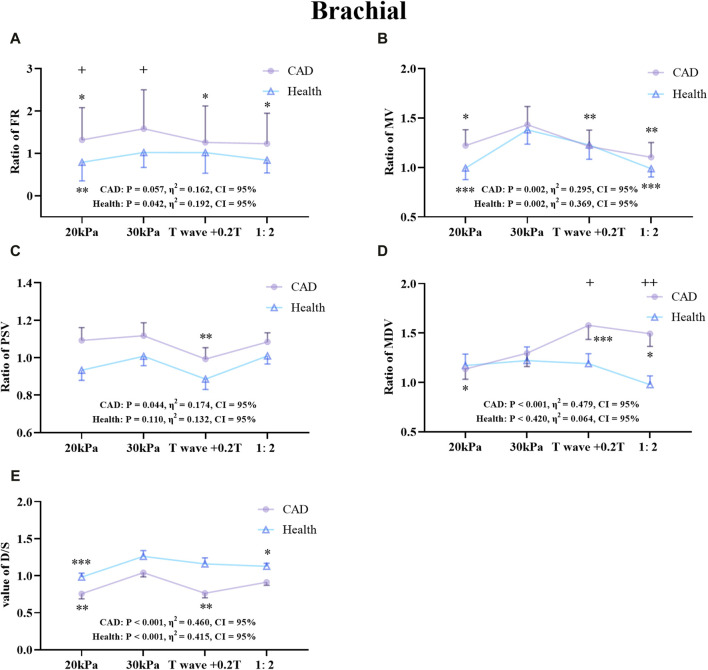
Comparison of the ratio of FR **(A)**, MV **(B)**, PSV **(C)**, MDV **(D)**, and the D/S value **(E)** in the RB between the patient and healthy groups, *P < 0.05 EECP-P30 vs another EECP state, **P < 0.01 EECP-P30 vs another EECP state, ***P < 0.001 EECP-P30 vs another EECP state, ^+^ *P* < 0.05 CAD vs Healthy, ^++^ *P* < 0.01 CAD vs Healthy. Results were evaluated by repeated-measures ANOVA (among 4 modes), by independent samples t-test (among two groups).

**FIGURE 10:**
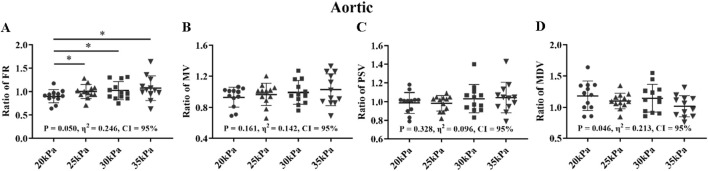
FR **(A)**, MV **(B)**, PSV **(C)**, MDV **(D)**, and ratio of EECP-P20 (20 kPa, T wave, 1:1), EECP-P25 (25 kPa, T wave, 1:1), EECP-P30 (30 kPa, T wave, 1:1), and EECP-P35 (35 kPa, T wave, 1:1) of AO in the healthy group, *P < 0.05 the EECP vs another EECP state. Results were evaluated by repeated-measures ANOVA (among four modes).

**FIGURE 11:**
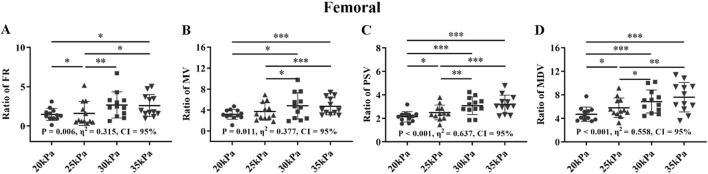
FR **(A)**, MV **(B)**, PSV **(C)**, MDV **(D)**, and ratio of EECP-P20 (20 kPa, T wave, 1:1), EECP-P25 (25 kPa, T wave, 1:1), EECP-P30 (30 kPa, T wave, 1:1), and EECP-P35 (35 kPa, T wave, 1:1) of RF in the healthy group, *P < 0.05 the EECP vs another EECP state, **P < 0.01 the EECP state vs another EECP state, ***P < 0.001 the EECP vs another EECP state. Results were evaluated by repeated-measures ANOVA (among 4 modes).

**FIGURE 12:**
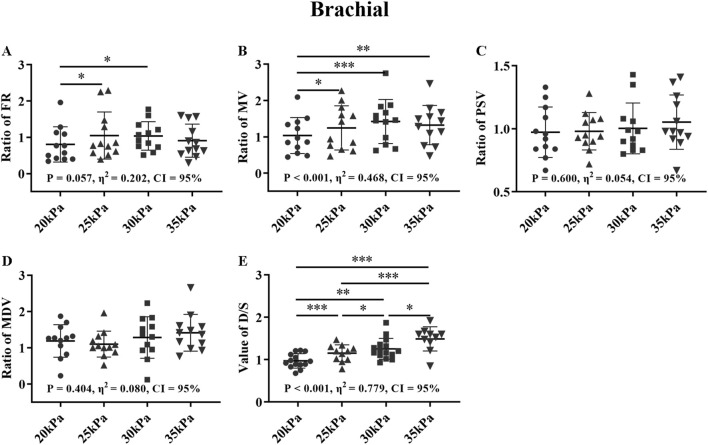
FR **(A)**, MV **(B)**, PSV **(C)**, MDV **(D)**, and ratio of EECP-P20 (20 kPa, T wave, 1:1), EECP-P25 (25 kPa, T wave, 1:1), EECP-P30 (30 kPa, T wave, 1:1), and EECP-P35 (35 kPa, T wave, 1:1) of RB and the D/S value **(E)** in the healthy group, *P < 0.05 the EECP state vs another EECP state, **P < 0.01 the EECP vs another EECP state, ***P < 0.001 the EECP vs another EECP state. Results were evaluated by repeated-measures ANOVA (among 4 modes).

The effects of different EECP modes on the hemodynamic parameters of the AO, RF, and RB were compared between the patient and healthy groups (Figures 7–9). The results of Figures 7–12 are described by letter labeling, with the same letter indicating no significant difference and without the same letter indicating a significant difference.

Figure 7 shows that there was a significant difference in the trends and values of hemodynamic parameters of the AO between the patient and the healthy groups under different EECP modes. For the FR and MDV of the two groups, EECP-P30 is significantly higher than EECP-P20. In the MV and PSV of the two groups, EECP-P30 was significantly higher than EECP-P20 and EECP-Freq. Specifically, FR in both groups and the PSV of CAD patients exhibited large effect sizes.

Figure 8 shows the trends and values of the hemodynamic parameters of the RF in the patient and healthy group under different EECP modes. For the FR of the two groups, EECP-P30 was significantly higher than EECP-P20, while in the PSV of the two groups, EECP-P30 was significantly higher than EECP-P20 and EECP-Freq. For the MV of the healthy group, EECP-P30 was significantly higher than the other three modes, while for CAD patients, EECP-P30 was significantly higher than EECP-P20 and EECP-Freq. For the MDV of the healthy group, EECP-P30 was significantly higher than EECP-P20 and EECP-Freq, while for the MDV of CAD patients, EECP-P30 was only significantly higher than EECP-P20. Notably, MV, PSV, and MDV in both groups as well as FR in CAD patients showed large effect sizes.

Figure 9 shows the trends and values of the hemodynamic parameters of the RB in the patient and healthy group under different EECP modes. For FR, EECP-P30 in CAD patients was significantly higher than the other three modes, while in healthy subjects EECP-P30 was only significantly higher than EECP-P20. For MV, EECP-P30 in CAD patients was significantly higher than other three modes, while in healthy subjects, EECP-P30 was significantly higher than EECP-P20 and EECP-Freq. For PSV, EECP-P30 in CAD patients was significantly higher than EECP-Time. The MDV of EECP-P30 in CAD patients was significantly higher than EECP-P20 but lower than EECP-Time and EECP-Freq. However, there was no significant difference in the comparison of EECP-P30 with different modes of PSV and MDV. For the D/S value, EECP-P30 in CAD patients was significantly higher than EECP-P20 and EECP-Time, while it was significantly higher in the healthy group than EECP-P20 and EECP-Freq. Additionally, FR, MV, and D/S in both groups, along with PSV and MDV in CAD patients, demonstrated large effect sizes.

### 3.3 Comparison of the different counterpulsation pressures in health group

Figures 10–12 show the effects of counterpulsation pressure on the hemodynamic parameters of the AO, RF, and RB for the healthy group among four states with only changed counterpulsation pressure (20 kPa, 25 kPa, 30 kPa, 35 kPa, respectively), T wave, 300 ms, 1:1.

For the FR of AO, 25 kPa, 30 kPa, and 35 kPa were significantly higher than 20 kPa (*P < 0.05*) (Figure 10). For the MV, PSV, and MDV of AO, there was only significantly among the four modes. The FR, MV and MDV of the AO exhibited large effect sizes, followed by the PSV of the AO with a medium effect size.

For the FR and MV of the RF, there were significant differences between 30 kPa, 35 kPa, pr 25 kPa, and 20 kPa, but no significant differences between 30 kPa and 35 kPa with 20 kPa and 25 kPa (Figure 11). For the PSV and MDV of the RF, there were significant differences between 30 kPa, 35 kPa with 25 kPa, 20 kPa, and 25 kPa with 20 kPa, but no significant differences between 30 kPa and 35 kPa. The four parameters of the RF all exhibited large effect sizes.

In Figure 12, for the FR of the RB, 25 kPa and 30 kPa were significantly higher than 20 kPa, and for the MV of the RB, 25 kPa, 30 kPa and 35 kPa were significantly higher than 20 kPa (*P < 0.05*), while for both of them there were no significant difference between 25 kPa, 30 kPa, and 35 kPa. In particular, in the comparison of D/S, all four pressures have significant differences from each other (*P < 0.05*). There was no significant difference for the PSV and MDV of the RB. The FR and MV of the RB and D/S exhibited large effect sizes, while the MDV of the RB showed a medium effect size.

### 3.4 0D-1D hemodynamic simulation and comparison

Meanwhile, we conducted population-based simulations based on the 0D-1D cardiovascular system model to explore the trends of blood flow and blood flow velocity in the AO, RF, and RB under different EECP modes in the hemodynamic simulation model. First, we optimized the coupled the coupling coefficients based on the flowchart in Figure 2B. In this study, the coefficient $c_{d}^{T\;wave+0.2T}$ was optimized to be 103.86 ${\left(cm/s\right)}^{2}/\sqrt{\left(kg/cm\right)}$ .

**FIGURE 13:**
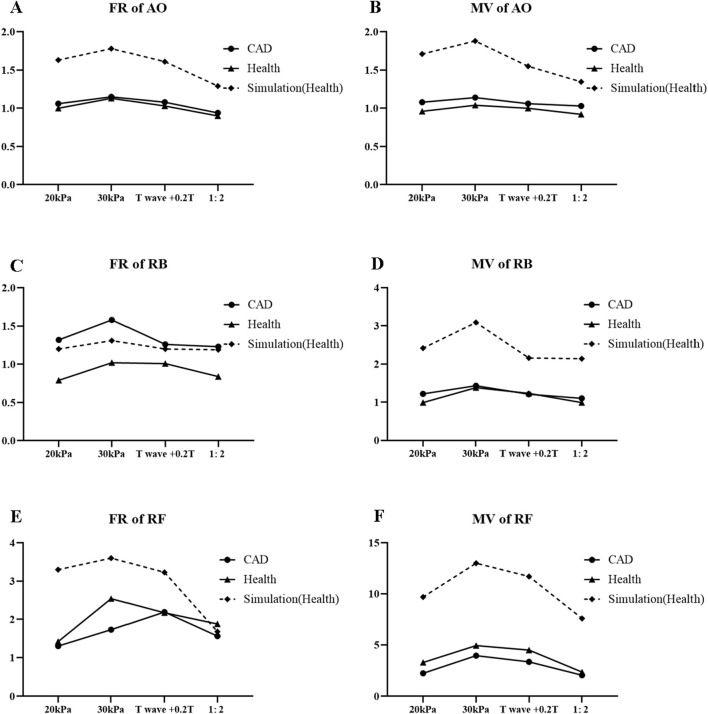
FR **(A)** and MV **(B)** of AO, FR **(C)** and MV **(D)** of RB, FR **(E)**, and MV **(F)** of RF for different EECP modes.

The simulation results of the 0D-1D cardiovascular model and comparison with clinical results for different EECP modes are shown in Figure 13. Despite the differences of exact values between the simulation and clinical results, the trends of simulation results under different EECP modes were generally closely aligned with clinical measurements. The simulation results also showed that the FR and MV of the AO, RF, and RB in EECP-P30 were higher than those of the other three models.

## 4 Discussion

In this study, the hemodynamic effect of EECP parameters was explored, including counterpulsation pressure, start time, and counterpulsation frequency; these were varied to conduct a clinical trial to analyze the changes of hemodynamic parameters in CAD patients as well as make comparisons with healthy subjects and population-based hemodynamic simulations. Higher counterpulsation pressure, T wave start time, and 1:1 counterpulsation frequency improved blood flow velocity and blood flow. The simulation results under different EECP modes were generally closely aligned with clinical measurements.

### 4.1 Counterpulsation pressure increases blood flow and velocity

When counterpulsation pressure was increased, there was a significant effect on the blood flow rate and peak systolic velocity enhancement in the aorta of CAD patients. A study found that EECP significantly increased diastolic and mean blood pressure and lowered systolic blood pressure in the central aorta, thus indirectly increasing blood flow to the heart (Michaels et al., 2002). Thus, the increase in FR with the increased counterpulsation pressure may be related to the induced higher diastolic blood pressure and lower cardiac afterload, resulting in the higher cardiac output. In addition to increased cardiac output in the acute response to counterpulsation pressure, other studies have also found that the improvement of myocardial perfusion from the long-term effects of EECP is related to the promotion of angiogenesis (Wang et al., 2023; Wu et al., 2006). Immunohistochemical staining and reverse transcriptase PCR analysis have shown that elevated levels of vascular endothelial growth factor (VEGF) expression are associated with the formation of new blood vessels (Luo et al., 2009). In animal studies, it was found that EECP treatment may improve myocardial perfusion by increasing angiogenesis (Xu et al., 2023). This mechanism of endothelial growth promotion related to the higher blood flow velocity may be a potential factor in the improvement of ischemic disease with long-term EECP therapy.

Increased counterpulsation pressure also significantly improved peripheral arterial (RB and RF) blood flow and blood flow velocity in CAD patients. Consistent with Zhang et al. (2021) and Du et al. (2023), EECP can significantly increase the lower limb blood flow and blood flow velocity in time, and is sensitive to pressure changes. In the case of higher counterpulsation pressure, it is due to more blood flow ejected from the heart during systole and more blood flow to the peripheral arteries. Meanwhile, because counterpulsation pressure applies directly on the lower limbs, the increase in blood flow of the femoral artery is greater than the increase in the brachial artery. In addition to the acute effects of counterpulsation pressure, studies have also found that EECP has long-term benefits for the upper limbs which increase skeletal muscle resistance to endothelium-dependent vasodilation. This may have improved peripheral arterial blood perfusion in the upper limb (Avery et al., 2014; Werner et al., 2007).

The counterpulsation effects of 20, 25, 30, and 35 kPa in the healthy group were compared. The FR of the AO in the healthy group showed a rise and then no change with increasing pressure. However, the effect of counterpulsation pressure on MV, PSV, and MDV did not change significantly. Meanwhile, the peripheral arteries (RB, RF) also showed a significant rise at 20–30 kPa and no significant difference at 30–35 kPa in RF, and a significant rise at 20–25 kPa and no significant difference at 25–35 kPa in RB. It may be that 25–30 kPa is the plateau pressure for the improvement of blood flow and that excessive increase in pressure does not have a significant effect. Unusually, PSV and MDV in the RB did not change significantly after increasing counterpulsation pressure, which may still be related to the direct action of the EECP device on the lower limbs.

### 4.2 Start time has a greater impact on peripheral arteries

With the delayed start time of counterpulsation, we found a significant decrease in the blood flow rate and flow velocity of the RB and flow velocity of the RF in CAD patients, but no significant difference was found in blood flow in the AO. This may be because the delayed start time may overlap the diastolic period with the EECP action time, which reduces the MV of the RF compared with the T-wave start time. However, the PSV of EECP-Time was slightly higher than that of EECP-P30, which was opposite to the effect of FR, MV, and MDV in the RF. It might be because the start time delay was 20% of the cardiac cycle; taking a cardiac cycle of 0.8 s as an example, the delay was 0.16 s. This is similar to the transit time of the aorta to the femoral artery, which may cause the EECP action to overlap with the arrival of the blood flow from aorta, so the femoral artery PSV value reaches its maximum at EECP-Time.

### 4.3 Counterpulsation frequency offers new clinical directions for some patients

When counterpulsation frequency is increased, the therapeutic effect is not as good as the counterpulsation frequency of 1:1. This is because the lower extremities are squeezed for a shorter time and the heart releases less blood flow during the same time. However, the hemodynamic parameters in the relative rest state are still improved, and this increase in the counterpulsation frequency can provide clinical treatment of EECP in patients with arrhythmia or pressure intolerance. Changing the counterpulsation frequency also opens up a new direction for clinical treatment, which can explore multiple compression modes of multiple cardiac cycles.

Additionally, the trends in hemodynamic therapeutic effects are generally consistent between CAD patients and healthy subjects. This consistency supports the use of healthy subjects in future experiments to further explore these effects. Specifically, the impact of EECP mode variations is more pronounced in CAD patients.

### 4.4 Hints for developing a patient-specific treatment strategy

EECP with various counterpulsation pressures, start times, and counterpulsation frequencies induced different hemodynamic responses. The hemodynamic effect of EECP is not only the most direct way to evaluate its clinical efficacy but also the direct goal of its therapeutic improvement. On the whole, EECP-P30 (30 kPa, T wave, 1:1) had the most significant effect on improving hemodynamic parameters in patients. There was no point in pursuing higher pressure to improve the blood flow. In addition, 25 kPa is high enough to improve the blood flow for healthy subjects with low blood pressure. Therefore, the optimal counterpulsation pressure may differ for different subjects with different blood pressures.

The hemodynamic parameters in the RF were all higher in healthy subjects than in CAD patients in EECP states, but the trends were essentially the same in both groups. This may be due to the difference in age between healthy subjects and patients, with younger subjects having better blood circulation. In addition, in order to avoid the numerical difference caused by the influence of initial blood flow, we reduced this error by the ratio method and found that subjects with better blood circulation in the rest state also showed better results in the treatment effect of EECP. Therefore, a longer treatment course may be necessary for the therapeutic improvement of patients with poor circulation.

The simulation results under different EECP modes based on 0D-1D cardiovascular system model were generally closely aligned with clinical measurements. Our previous model validated the simulation of a single EECP mode, but the results of this study confirm the validity of this model across different EECP modes. The similar trends observed between the simulation and clinical results reflect the general effects of different EECP modes and further verify the accuracy of the model. The discrepancy between the clinical results and the simulation results for each EECP mode indicates the inter-individual variability. The combination of clinical experiments and model simulation allows a more comprehensive analysis of the role of EECP on blood flow redistribution. Clinical experiments are used to analyze the effects of different EECP parameters and validate subsequent model simulations. Model simulations prepare the way for the creation of virtual databases for a large number of experiments to obtain population-based strategies and allow for precision-based strategies through individual modelling.

### 4.5 Limitations

This study had some limitations. First, the sample size of each group was relatively small, which is a common limitation in clinical trials due to challenges such as high costs and time constraints. Additionally, the advanced age of clinical CAD patients introduces an unavoidable factor that necessitates careful consideration of age-related differences. Consequently, future experiments will emphasize subject classification, particularly through age-based grouping. Second, blood flow was measured only in three arteries using Doppler ultrasound. This approach is limited by challenges inherent in clinical trials, such as the complexity of measuring hemodynamic parameters at all sites of interest and the potential for experimental *in vivo* measurement errors. For example, errors in ultrasound measurements due to angle, relative position, and individual subject variability are unavoidable. Third, the hemodynamic effects of compression duration were not investigated in this study. Fourth, only the acute hemodynamic responses to EECP modes were analyzed.

## 5 Conclusion

This study details the effects of EECP counterpulsation pressure, start time, and counterpulsation frequency on hemodynamics parameters in the AO, RB, and RF of patients with cardiovascular disease and of healthy subjects. The hemodynamic simulations based on a 0D-1D cardiovascular system model were combined with the clinical results to verify each other. Our findings lead to the following conclusions.

(1) EECP with various counterpulsation pressure, start time, and counterpulsation frequency induced different hemodynamic responses. Thus, it is advisable to adjust these three parameters to achieve balance between therapeutic improvement and patient comfort.
(2) Counterpulsation pressure is the most direct way to improve aortic and peripheral arterial blood flow and velocity in patients. The most cost-effective pressure to increase arterial flow may be 30 kPa.
(3) Once the counterpulsation pressure is fixed, the start and ends time of counterpulsation should be changed to achieve optimal therapeutic effect.
(4) Increasing the counterpulsation frequency relatively reduces blood flow and blood velocity but still improves relative to the rest state and relieves discomfort during EECP; this can provide proper clinical treatment of EECP in patients with arrhythmia or pressure intolerance.
(5) Hemodynamic simulations based on the verified model prepare the way for the creation of virtual databases for a large number of experiments to obtain population-based strategies and then allow for precision-based strategies through individual modelling.

Overall, our comprehensive investigation underscores the benefits of the different EECP modes for blood flow improvement in patients with cardiovascular diseases and offers exciting avenues for further research and development in this field.

## Data Availability

The raw data supporting the conclusions of this article will be made available by the authors, without undue reservation.
